# An Analysis of the Prevalence and Trends in Drug-Related Lyrics on Twitter (X): Quantitative Approach

**DOI:** 10.2196/49567

**Published:** 2024-12-30

**Authors:** Waylon Luo, Ruoming Jin, Deric Kenne, NhatHai Phan, Tang Tang

**Affiliations:** 1 Department of Computer Science Kent State University Kent, OH United States; 2 College of Public Health Kent State University Kent, OH United States; 3 Department of Data Science New Jersey Institute of Technology Newark, NJ United States; 4 School of Media and Journalism Kent State University Kent, OH United States

**Keywords:** Twitter (X), popular music, big data analysis, music, lyrics, big data, substance abuse, tweet, social media, drug, alcohol

## Abstract

**Background:**

The pervasiveness of drug culture has become evident in popular music and social media. Previous research has examined drug abuse content in both social media and popular music; however, to our knowledge, the intersection of drug abuse content in these 2 domains has not been explored. To address the ongoing drug epidemic, we analyzed drug-related content on Twitter (subsequently rebranded X), with a specific focus on lyrics. Our study provides a novel finding on the prevalence of drug abuse by defining a new subcategory of X content: “tweets that reference established drug lyrics.”

**Objective:**

We aim to investigate drug trends in popular music on X, identify and classify popular drugs, and analyze related artists’ gender, genre, and popularity. Based on the collected data, our goal is to create a prediction model for future drug trends and gain a deeper understanding of the characteristics of users who cite drug lyrics on X.

**Methods:**

X data were collected from 2015 to 2017 through the X streaming application programming interface (API). Drug lyrics were obtained from the Genius lyrics database using the Genius API based on drug keywords. The Smith-Waterman text-matching algorithm is used to detect the drug lyrics in posts. We identified famous drugs in lyrics that were posted. Consequently, the analysis was extended to related artists, songs, genres, and popularity on X. The frequency of drug-related lyrics on X was aggregated into a time-series, which was then used to create prediction models using linear regression, Facebook Prophet, and NIXTLA TimeGPT-1. In addition, we analyzed the number of followers of users posting drug-related lyrics to explore user characteristics.

**Results:**

We analyzed over 1.97 billion publicly available posts from 2015 to 2017, identifying more than 157 million that matched drug-related keywords. Of these, 150,746 posts referenced drug-related lyrics. Cannabinoids, opioids, stimulants, and hallucinogens were the most cited drugs in lyrics on X. Rap and hip-hop dominated, with 91.98% of drug-related lyrics from these genres and 84.21% performed by male artists. Predictions from all 3 models, linear regression, Facebook Prophet, and NIXTLA TimeGPT-1, indicate a slight decline in the prevalence of drug-related lyrics on X over time.

**Conclusions:**

Our study revealed 2 significant findings. First, we identified a previously unexamined subset of drug-related content on X: drug lyrics, which could play a critical role in models predicting the surge in drug-related incidents. Second, we demonstrated the use of cutting-edge time-series forecasting tools, including Facebook Prophet and NIXTLA TimeGPT-1, in accurately predicting these trends. These insights contribute to our understanding of how social media shapes public behavior and sentiment toward drug use.

## Introduction

### Background

The prevalence of drug and alcohol references in popular music has exhibited a significant increase over the years [1-3]. The impact of lyrical drug content by musicians can significantly influence the mental health and overall well-being of both adolescents and adults [4]. Drug lyrics can be traced back to the emergence of The Beatles in the 1960s. Notably, The Beatles’ renowned hit song, “Lucy in the Sky with Diamonds,” is widely believed to contain an allusion to lysergic acid diethylamide [5]. Since then, rock music has gradually become more explicit in its lyrical depictions of sex, drugs, and violence [4,6]. In the past decade, the inclusion of explicit drug lyrics in popular songs across various genres has experienced exponential growth. According to the Genius database, the number of popular songs related to drugs has increased by 1625% from 1999 to 2017. Furthermore, nearly 50% of the top 40 songs in 2016 contained references to drugs and alcohol [7].

**Figure 1:**
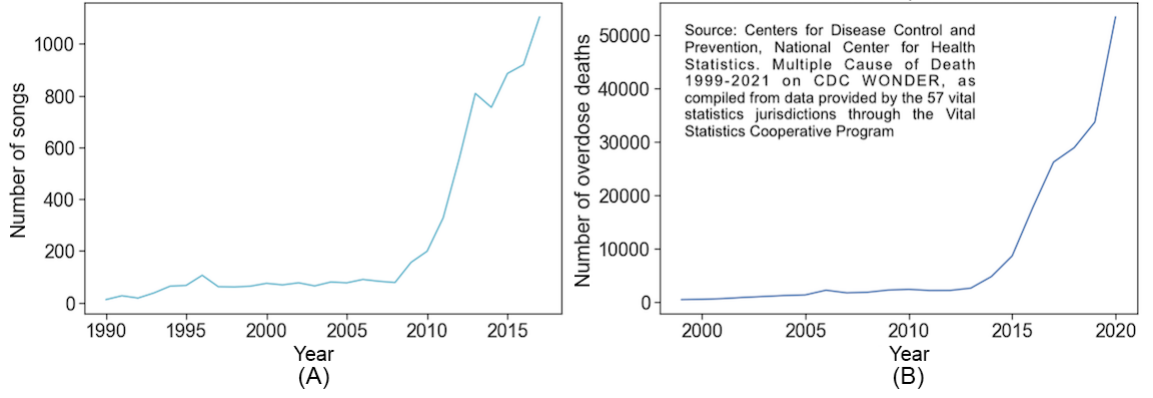
Comparison of trends in drug-related songs and opioid overdose deaths, (A) number of substance use-related songs from 1990 to 2019, based on data from the Genius.com lyrics database and (B) number of overdose deaths involving opioids, including fentanyl, in the United States from 1999 to 2020, based on data from the Centers for Disease Control and Prevention.

Simultaneously, the United States is grappling with an escalating epidemic of prescription and illicit drug use. Opioid use disorder and overdoses incur thousands of premature deaths and billions of dollars in economic losses annually [8,9]. The number of opioid-related deaths surged from approximately 5000 to 30,000 in 2015 alone [10]. Furthermore, between 2014 and 2015, fatalities resulting from synthetic opioids like fentanyl increased by nearly 75% [11]. According to data from the Centers for Disease Control and Prevention’s National Center for Health Statistics, overdose deaths involving opioids, including fentanyl, escalated by 6308% from 1999 to 2017 [11]. Figure 1 displays charts illustrating comparable exponential growth patterns, albeit on different scales.

Drug use content on social media has gained prominence as a burgeoning mode of communication [12,13]. The prevalence of user-generated drug abuse content on social media platforms has been well-documented [14]. These extensive datasets of online drug-related communications have emerged as a primary source, allowing researchers to interpret human behaviors, identify patterns, and analyze evolving drug cultures [15,16].

The motivation behind why certain Twitter (subsequently rebranded X) users post drug lyrics can vary and is influenced by a combination of individual factors, social dynamics, and contextual factors. Some X users may share drug lyrics as a form of self-expression or artistic appreciation. They may resonate with the lyrical content, find it relatable to their experiences, or enjoy the creativity and wordplay. Popular culture, including music and media, often glorifies drug use and references. X users may be influenced by these cultural norms and trends, seeking to align themselves with particular social identities or portray a specific image by sharing drug lyrics. Posting drug lyrics may serve as a means for individuals to connect with like-minded individuals or subcultures with similar experiences or interests. It can create a sense of belonging and camaraderie among users who relate to the themes and emotions depicted in these lyrics. Some users may post drug lyrics to gain attention, shock, or provoke reactions from their followers or the broader X community. They may seek validation and controversy or simply thrive on the engagement and responses generated by controversial or provocative content. For some users, sharing drug lyrics may serve as a form of catharsis or emotional outlet. It can provide an avenue for expressing and processing their struggles, emotions, or experiences related to substance abuse or addiction.

The reasons behind posting drug lyrics on X can be complex and multifaceted, varying from individual to individual. Examining and investigating the specific driving factors and psychological dynamics behind such behavior necessitate further research and analysis.

Various studies and publications relate drug lyrics in music to substance use content on social media. These studies probe the influential role of music in shaping individuals’ identities and behaviors, and the direct impact musicians have on their followers [17].

Studies show that there is an association between music preferences and drug abuse patterns, focusing on heavy metal music as an example. Research from the 1990s suggests that fans of heavy metal music were more likely to engage in risky behaviors, including drug abuse [18-20]. Studies analyzing drugs in popular music lyrics provide further evidence of the prevalence of drug, alcohol, and tobacco references. Primack’s analysis of Billboard’s most popular songs in 2005 revealed that one-third of the songs portrayed drugs, with rap and country genres having the highest representation [1]. Similarly, a study on alcohol and tobacco impressions from popular YouTube music videos demonstrated that these videos generate billions of gross impressions of drug content, with adolescents being particularly susceptible to such exposure [21].

Owing to the burgeoning advancements in machine learning methodologies in various domains [22-26], significant strides have been made in enhancing the predictive accuracy and efficiency of various computational models [27,28]. Researchers have used machine learning algorithms to detect and interpret drug abuse content on platforms like X. Studies using support vector machine [29], long short-term memory (LSTM) [30], and other machine learning methods have achieved varying levels of accuracy in classifying drug abuse posts [31]. Real-time drug abuse detection software using these algorithms has been developed to monitor current drug trends on social media [32,33].

A study found a significant correlation between the frequency of cocaine references in music lyrics and cocaine use incidence and mortality rates. This suggests that drug mentions in popular music could indicate epidemiological trends [34]. Among the individuals worth highlighting is Mac Miller, a renowned American singer and producer whose lyrics garnered considerable attention in the realm of drugs. Between 2015 and 2017, Mac Miller’s lyrics were posted a remarkable 1808 times, indicative of their influence and resonance with the audience. Tragically, in September 2018, Mac Miller succumbed to the perils of “mixed drug toxicity,” stemming from a lethal combination of cocaine, fentanyl, and alcohol [35].

Similarly, musician Juice WRLD experienced a substantial presence of his lyrics on X, with 298 posts recorded during the same timeframe. Regrettably, in December 2019, Juice WRLD met an accidental overdose of codeine and oxycodone, resulting in his untimely demise [36]. A larger X dataset and a complete lyrics database may provide more robust evidence of a correlation between drug lyrics on X and the mortality of artists associated with the lyrics.

In this section, an overview of research conducted on drug lyrics in music and substance use content on social media is provided, highlighting the influence of music on individuals’ behaviors, the prevalence of drug references in popular music, and the role of social media in facilitating the spread of drug-related content. However, none of these studies have explored the overlapping domain of user-generated drug lyrics references on social media. While the dataset used in this study comprises posts from 2015 to 2017, our study’s primary novelty lies in identifying and defining a new subcategory of X content: “tweets that reference established drug lyrics.” This categorization provides a unique lens through which to analyze social media’s influence on public behavior and sentiment related to drug use. Furthermore, this dataset may be an important factor in modeling the exponential increase in drug-related incidents, offering valuable insights for public health and safety interventions. To ensure the robustness and modernity of our analytical approach, we evaluated our results using the latest prediction technologies, including the Facebook Prophet time-series forecasting application programming interface (API) and the NIXTLA TimeGPT-1. These advanced tools demonstrated that the methodologies used can be effectively applied to historical and contemporary data, thereby contributing valuable knowledge to the ongoing discourse on the drug epidemic.

### Definition of Drug Lyrics

Drug lyrics refer to lyrics that mention drugs, including their use, possession, or dealing, either directly or indirectly. One of the objectives of this study is to identify posts pertaining to drug lyrics. To determine if a post contains drug lyrics, we apply the following criteria.

#### Authenticity

The lyrics must be established and documented in the lyrics database to ensure their authenticity.

#### Specificity

A minimum threshold of at least 3-word matches is required after excluding stop words to demonstrate the specificity of drug lyrics in posts.

**Figure 2:**
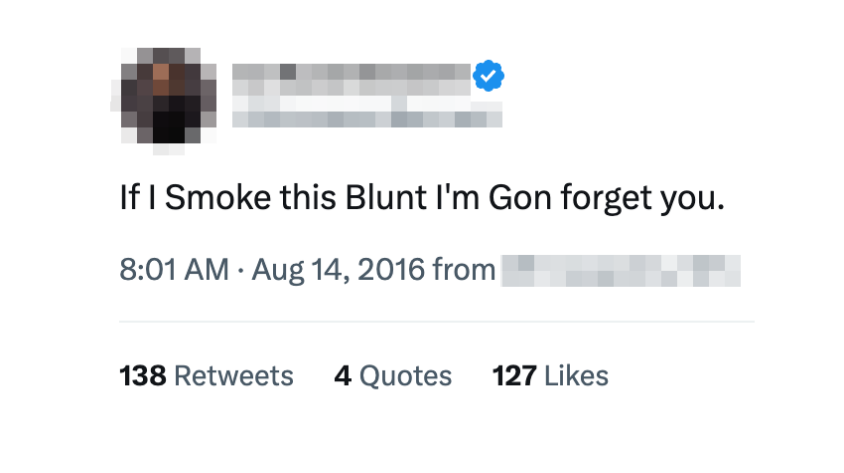
Example of a drug-related lyrics tweet by an internet influencer. This tweet references a lyric from Chief Keef’s song “Love No Thotties” and includes mention of drug use (“blunt”).

A post by an influencer, as shown in Figure 2, “[And] If I smoke this blunt[, girl,] I’m gon forget you.” from the song “Love No Thotties” by Chief Keef, is considered authentic due to its presence in the reference lyrics database provided by the Genius API and its demonstration of originality. However, a portion of the posts, such as “I smoke this blunt,” are not considered lyrics because they contain only 2 keywords, “smoke” and “blunt.”

## Methods

### Data Collection and Preprocessing

**Figure 3:**
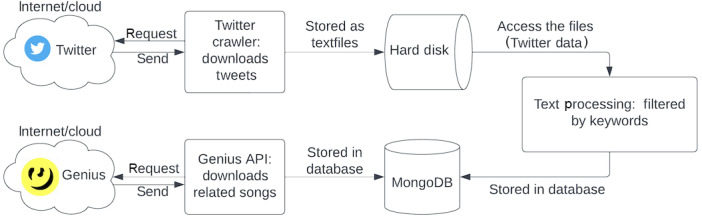
The diagram illustrates the 2 processes involved in data collection for the study. In the first process (top), tweets are downloaded from Twitter using a custom Twitter Crawler by the Twitter API. In the second process (bottom), the Genius API is used to retrieve lyrics of songs related to drug keywords from the Genius database.

Real-time X data was acquired using the X APIs through the X Crawler [33]. The dataset contains posts gathered from 2015 to 2017, spanning a total of 1.97 billion posts. These posts were organized into hourly batches and stored as text files. Before the analysis, a preprocessing step was performed, which involved filtering 190 keywords related to drugs. The selection of these keywords was based on a combination of “pharmaceutical” and “street” terms associated with drugs that have gained prominence in popular music [37]. The flow diagram of data collection is depicted in Figure 3. The process involved using the open-source Python library, “lyricsgenius,” in conjunction with the Genius API to procure songs that contain drug lyrics [38]. The selection was based on 17 prominent drug keywords, acquiring a substantial corpus of 13,066 documents in JSON format, which were subsequently stored in MongoDB. Out of this collection, 10,292 documents featured lyrics composed in English, while 2003 documents predominantly contained lyrics in non-English languages such as Spanish, French, and Russian. In addition, 771 documents comprised scripts from documentaries, interviews, and other textual sources unrelated to lyrics. Our investigation exclusively focuses on the analysis of drug abuse lyrics written in English.

A series of preprocessing steps were conducted to filter and process the posts. Initially, the posts were tokenized. Then, the resulting tokens were compared against the predefined drug keywords. To enhance the search quality and efficiency, lemmatization was applied to both the tokenized posts and the drug keywords. The lemmatization process involved reducing inflectional forms and derivationally related forms of words to a common base form [39]. For this purpose, the WordNetLemmatizer from the Natural Language Toolkit Python library was used.

To identify drug keywords in the collected posts, tokens were compared against those keywords using the fuzzy string-matching API from the FuzzyWuzzy Python library. This API returns a match score for each token and drug keyword pair. The match score, normalized to a value between 0 and 1, is computed based on the Levenshtein distance between 2 strings, which counts the minimum number of single-character edits required to transform 1 string into another. A drug-related post is detected if tokens match drug keywords with a fuzzy match score of 0.95 or higher. This threshold allows for a small variance in tokens when matching with drug keywords. Over 157 million posts matched the drug keywords. The processed posts were then stored in JSON format in a MongoDB database.

The processed posts were iterated and compared against the lyrics present in our lyrics database to identify posts containing lyrics. This comparison was performed using a custom API called LyricsMiner, specifically designed for this purpose. The goal was to identify instances of drug lyrics within the posts.

As posted lyrics may contain misspelled words, twisted words, or word contractions, the references to lyrics on X that we aim to find may not match the lines of text in our reference database. Instead of requiring an exact match, a similarity search is required. For this purpose, we used LyricsMiner, a specialized text-matching API explicitly designed to identify drug lyrics by using similarity search techniques.

### LyricsMiner

The LyricsMiner is a custom API that identifies drug lyrics from our collected X data. Text preprocessing includes a series of steps that involve tokenization. Posts are broken down into individual words and changed to lowercase, while punctuation marks are removed. Posts with less than 3 words, special characters, hyperlinks, usernames, emojis, and all stop words are excluded from the post. In addition, a stemming process is used, whereby words are reduced to their root form. A search query is constructed based on the tokens to streamline the matching process, enabling the retrieval of lyrics from the lyrics database. The query returns possible lyric matches that align with the tokenized texts. A local sequence alignment technique, as described by Smith and Waterman [40], is used to identify potential matches. The detailed implementation is explained in Multimedia Appendix 1. The local sequence alignment algorithm is used for similarity searches, accommodating cases where posts and lyrics may be similar but not identical. Each comparison yields a matching score ranging from 0 to 1, where a score of 0 indicates a complete mismatch and a score of 1 reflects a perfect match. The metadata is updated in the post database in MongoDB if drug lyrics are detected. The metadata includes the portion of original reference lyrics, artists, songs, and their release year.

**Figure 4:**
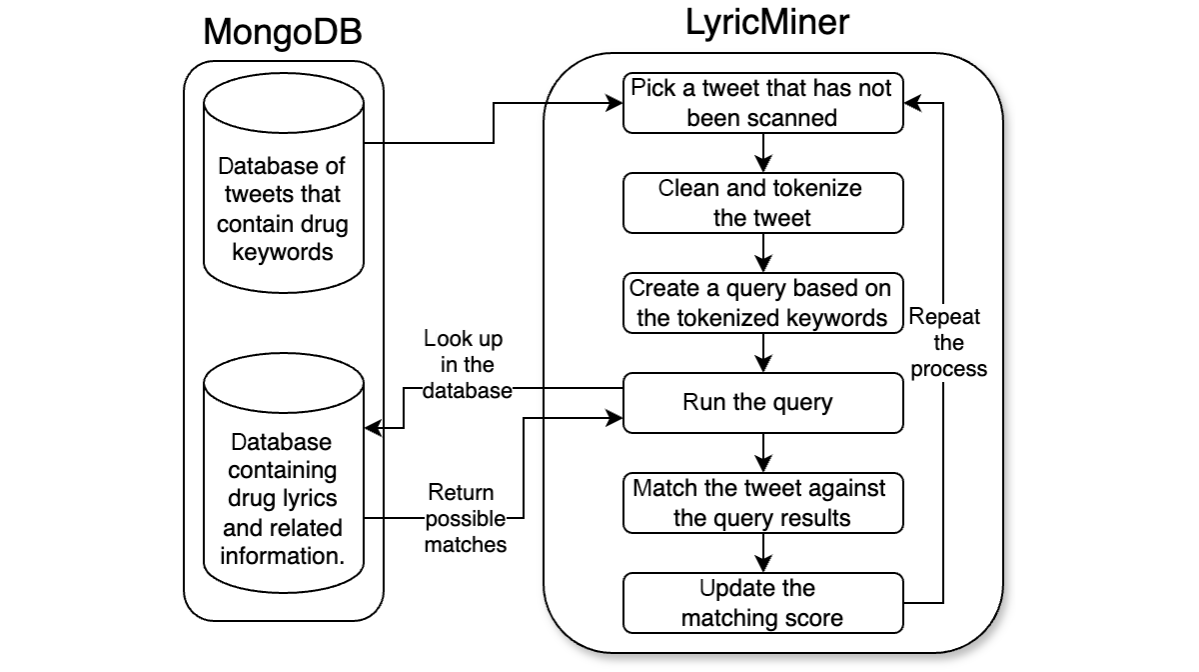
Flow diagram of the LyricMiner application processing interface for identifying drug-related lyrics in posts. The diagram illustrates the workflow of the LyricMiner system used to match tweets containing drug-related keywords with corresponding lyrics from a song database.

The process commences by retrieving positive posts from the database, using a buffer size of 200, which are subsequently iterated through one by one. The search process continues iteratively, with the program progressing to the next post in the queue until all posts have been scanned, as depicted in Figure 4. The LyricsMiner API exhibited an average scanning rate of 20 (SD 3.88) posts per second, and the total time required to process all the posts amounted to approximately 1460 hours. The source code for LyricsMiner is available in our GitHub repository [41].

### Analysis Methods

#### Random Sampling

A random sampling technique was applied to test the accuracy of the LyricsMiner. Random sampling is a method where each element in a set has an equal chance of being selected to form a sample, ensuring an unbiased representation of the whole group. A thousand posts with a matching score of 0.5 and above were randomly selected from the database and manually reviewed by our team. False positive drug lyrics were grouped by song, manually reviewed, and eliminated based on the definition of drug lyrics.

#### Cross-Analysis

Cross-analysis is a method that involves examining the relationships or interactions between different variables, typically across multiple datasets or categories, to identify patterns or correlations. We performed a cross-analysis with the Billboard top 100 year-end chart to gauge the popularity of the original references. The Billboard top 100 is a music chart published weekly by Billboard magazine, ranking the 100 most popular songs based on factors such as radio airplay, digital sales, and streaming data. Billboard year-end charts are collected through the Python APIs for Billboard. The identified drug-lyrics posts were matched against yearly Billboard charts.

#### Time-Series Analysis

Time-series analysis is a statistical technique that analyzes time-ordered data points to understand trends and variations and forecast future events based on historical patterns. It enables us to examine temporal trends in post volume and drug lyric matches from 2015 to 2017. The time-series drug lyrics are prepared by batching the identified drug lyrics in posts over a period of months. Then, we applied a linear regression model, the Prophet model, and TimeGPT-1 to the data.

The linear regression model is a statistical method that predicts future values by establishing a linear relationship between the dependent variable and 1 or more independent variables based on historical data. Prophet is an open-source forecasting tool Facebook’s Core Data Science team developed [42]. It is designed to forecast time-series data with seasonal components and historical data. Prophet makes time-series forecasting accessible and interpretable, emphasizing simplicity and automation. The model has 3 main components: trend, seasonality, and holidays. [43,44] We also leveraged a generative pretrained transformer for the time-series model TimeGPT-1 [45] to predict future outcomes. TimeGPT-1, developed by NIXTLA [46], is easy to use through public APIs and requires minimal programming tasks. The APIs allow us to overcome the need for machine learning teams to create our prediction models.

The data are split into training and testing sets. The training data fits a model where the target variable is the time-series value. The test dataset is used to test the accuracy of the trained model. Root-mean-squared error (RMSE) and *R*^2^ values are presented as the model’s performance metrics. RMSE measures the average magnitude of the errors between the predicted and actual values, showing how well the model’s predictions match the observed data. The R^2^ value indicates how well the data fits the model.

#### Histogram Distribution

A histogram distribution is a statistical representation of data that groups numerical data into bins, showing the frequency of data points within each bin to illustrate the underlying distribution of the data. A histogram distribution method is used to analyze one of the user characteristics, the number of followers of X users who posted drug lyrics. We first identified the minimum and maximum values in the dataset to determine the range. This range was then divided into 100 equal intervals or bins, each representing a specific range of values. We proceeded by counting the number of data points, the number of followers, that fall into each bin.

### Ethical Considerations

In conducting research on X and drug abuse-related posts, we adhered to stringent ethical guidelines to protect individuals’ privacy and confidentiality. All data included in the study were fully deidentified, with all personally identifiable information, such as usernames, real names, and other identifying details, being thoroughly anonymized during the data collection process. Furthermore, we ensured compliance with X’s terms of service and adopted transparent and respectful data handling methods. These measures allowed us to maintain our research’s integrity and the privacy and confidentiality of the individuals whose posts were analyzed.

## Results

### Twitter Datasets

A dataset comprising over 1.97 billion posts collected between 2015 and 2017 was obtained using the X Crawler and Streaming API [33]. A total of 1.976 billion posts are organized into hourly batches. Following the filtering process, 157 million posts were identified that matched with drug keywords, out of which 150,746 were found to contain lyrics references. Further details can be found in Table 1. Due to technical limitations, specific X data were not captured. A breakdown of the total number of hours, available hours, and missing hours can be found in Table 2.

**Table 1:** Summary of post data, processed posts, and lyrics matches from 2015 to 2017.

Post categories	Posts in 2015, n	Posts in 2016, n	Posts in 2017, n	Total, n
Original posts	635,515,709	553,623,151	787,108,543	1,976,247,403
Processed posts	51,019,894	49,057,982	57,489,144	157,567,020
Lyrics matches	61,267	52,279	37,200	150,746

**Table 2:** Availability of Twitter data by year (2015-2017). The data are organized into hourly batches.

Year	Total days, n	Available days, n	Missing days, n	Total hours, n	Available hours, n	Missing hours, n
2015	365	328	37	8760	7796	964
2016	366	309	57	8784	7421	1363
2017	365	339	26	8760	8106	654

### Popular Drugs in Lyrics on Twitter Space

**Figure 5:**
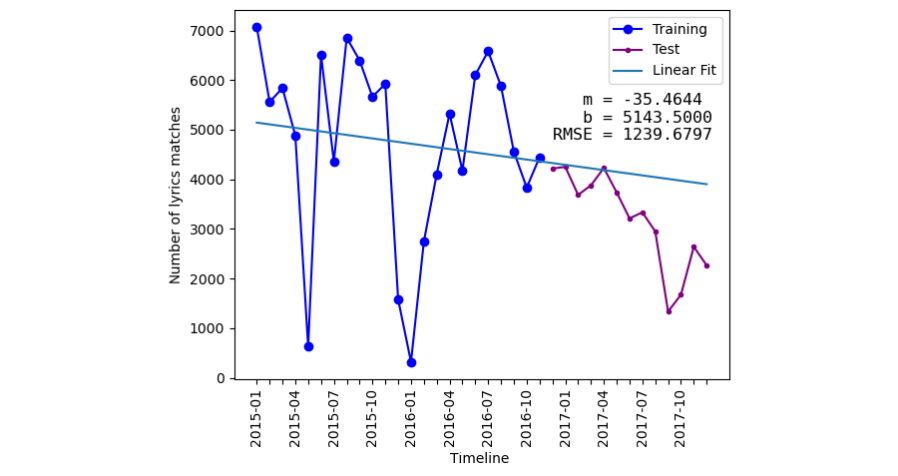
The plot depicts the results of a linear regression model applied to the number of drug lyrics matched on Twitter between 2015 and 2017. The blue line represents the training data, while the purple line shows the test data, with the linear fit applied across both. RMSE: Root mean squared error.

Identified drug lyrics posts are categorized into 2 main groups, psychoactive drugs and others, as listed in Table 3. Cannabinoids, stimulants and hallucinogens, and opioids are classified as psychoactive drugs. Liquor, wine, and xan are listed as others. The drugs most referenced in the lyrics on X space are classified into 3 distinct categories: cannabinoids, opioids, stimulants, and hallucinogens. As shown in Figure 5, results indicate that cannabinoids, particularly marijuana, were the most referenced drug category, accounting for a total of 42,430 posts within the lyrics analyzed. Stimulants and hallucinogens were the second most referenced drug categories, with 23,949 posts, respectively. Opioids were mentioned in 12,958 posts. Overall, the findings suggest that cannabinoids are the most mentioned drugs within lyrics shared on X.

**Table 3:** Most frequent drugs are categorized into 4 groups: cannabinoid, stimulant and hallucinogen, opioid, and others. The term “Dope” is slang that can refer to various drugs but is most commonly used to denote marijuana or heroin.

Drug				Posts, n
**Psychoactive drugs**				
	**Cannabinoid (n=42,430)**			
		Weed	23,031	
		Blunt	13,319	
		Joint	2549	
		Dank	1830	
		Marijuana	1619	
		420	44	
		Mary Jane	38	
	**Stimulant and hallucinogen (n=23,949)**			
		Molly	9984	
		Cocaine	5096	
		Coke	4007	
		Powder	2865	
		Cigarette	1910	
		Crack	54	
		Mushroom	26	
		Ketamine	7	
	**Opioid (n=12,958)**			
		Dope	11,958	
		Heroin	811	
		Oxy	189	
**Others (n=8779)**				
	Liquor		4524	
	Wine		2795	
	Xan		1460	

Cross-referencing posts with Billboard charts provides insights into drug lyrics’ popularity and cultural impact in popular music. Our analysis identified which of the top 20 most posted drug lyrics each year appeared on the Billboard chart. An examination of the Billboard top 100 charts indicates that 7 songs featuring drug abuse lyrics were posted in 2015, 3 in 2016, and four in 2017. The most notable artists of drug lyrics being referenced are Drake, Future, G-Eazy, and Rob $stone. The details of the lyrics, songs, and artists are listed in Multimedia Appendix 2.

### Popular Drug Lyrics on Twitter

The study analyzed the top 20 most frequently quoted drug lyrics on X from 2015 to 2017. Our analysis included 38 artists, with 32 (84.21%) identified as male and 6 (15.79%) identified as female. Most of the quoted lyrics (91.83%) belonged to the rap or hip-hop genres, while 2 songs (4.08%) were categorized as rhythm and blues or soul, and 2 songs (4.08%) were categorized as pop or alternative.

Some prominent drug lyrics posts are discussed in this section. The phrase “Smoke good weed with a bad bitch” from the track “Good Weed Bad Bitch” by K CAMP was posted a total of 2721, 1109, and 383 times during the years 2015, 2016, and 2017, respectively. Similarly, the line “Double cup love, you the one I lean on” from “Best I Ever Had” by Drake witnessed consistent engagement on X, accumulating 546, 597, and 480 posts during the same period (2015-2017).

In 2016, the lyrics “Said she wanna roll with me, and smoke up all my weed” from the song “Chill Bill” by Rob $tone received the highest number of posts, 1901. The second most posted lyrics in the same year, “All she wanna do is smoke that broccoli,” were from the song “Broccoli” by DRAM, which accumulated 1327 posts.

The most posted drug lyrics “In New York I Milly Rock, [Hide it in my sock]” from the song “Magnolia” by Playboi Carti, released on April 14, 2017, garnered significant attention on X, accumulating over 3342 posts. The lyrics page of Genius recorded a substantial 2.6 million views for this song as of May 22, 2023, indicating its widespread popularity and cultural impact.

Furthermore, a similar variation of the lyrics emerged in the song “Bali” by 88GLAM, with the line “In New York I Milly Rock, My shooter tote a 30, he can't hide it in his sock.” The track was released on November 7, 2017, and has accumulated 281,600 views on the lyrics page of Genius as of May 22, 2023. Since “Magnolia” was released before “Bali,” all the counts go toward “Magnolia,” even though the phrase could be a partial match for “Bali.”

Similarly, the line “Neighbors think I’m sellin’ dope” or similar variations experienced notable activity on X, with 622 posts recorded in 2016 and 800 posts in 2017. An analogous phrase, “My mama say the neighbors think I’m selling drugs,” made its debut in the song “Bussin Juugs” by Gucci Mane, released in August 2012, accumulating 62,100 views on the lyrics page of Genius as of May 2023. However, it was not until a modified rendition of this line emerged in the song “Neighbors” by J. Cole, released in December 2016, that it gained substantial popularity, amassing 1.6 million views on the lyrics page of Genius as of May 2023.

Male artists predominantly perform most drug lyrics being posted. However, there are also female artists whose drug lyrics are being posted. In 2015, a line from “New Americana” by Halsey, “We are the new Americana, high on legal marijuana,” was posted 487 times. In 2017, a line from the song “MotorSport” featuring Nicki Minaj, “You don't want smoke with me, this is a laced blunt,” was posted 305 times.

### Prediction Model

We have evaluated the time-series data with 3 prediction models: linear regression, timeGPT-1, and Facebook Prophet. For the performance evaluation of various prediction models, all posts from 2015 through 2017 were batched by month and split into 66.7% training data and 33.3% test data. From the models, the RMSE and R^2^ values are calculated.

Linear regression has an RMSE of 1239.67 and R^2^ value of –0.91428. The linear regression model also yields *m* and *b* values. The *m* value corresponds to the dataset’s slope, while the *b* value represents the interception point with the y-axis. A negative *m* value of –35.4644, Figure 5 indicates a downward trend in drug lyrics posts over time.

**Figure 6:**
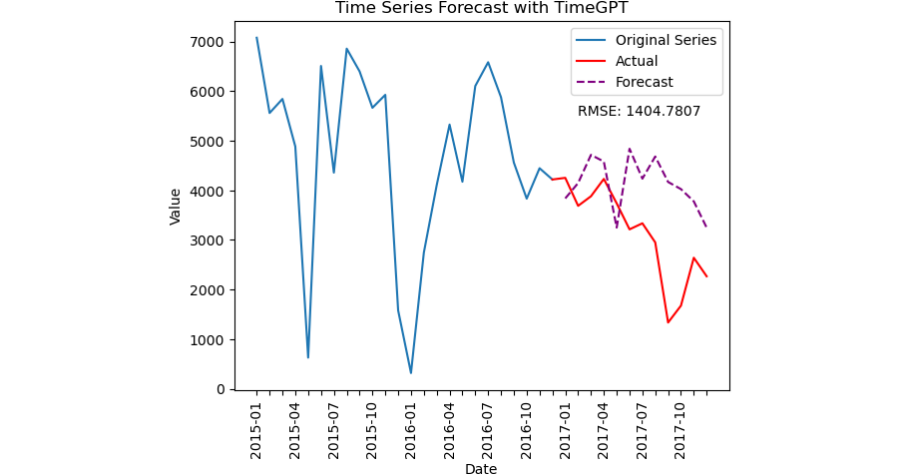
The plot illustrates the time series forecast for the number of drug-related lyrics on Twitter from 2015 to 2017 using the TimeGPT-1 model. The blue line represents the original series of actual data up until early 2017, while the red line shows the exact values for the test period. The dashed purple line represents the forecasted values generated by TimeGPT-1.

**Figure 7:**
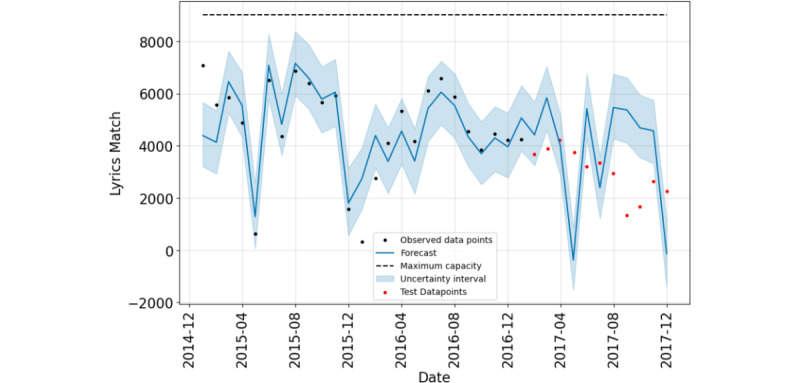
This plot shows the forecast of drug-related lyrics on Twitter from 2015 to 2017, generated by the Facebook Prophet model. The black dots represent the observed data points, while the solid blue line shows the forecast based on historical data. The light blue shaded area indicates the model’s uncertainty interval. The red dots represent test data points used to validate the model. The dashed horizontal line marks the maximum capacity threshold.

Facebook Prophet and TimeGPT-1 predict similar downward trends, as shown in Figures 6 and 7. Facebook Prophet showed an RMSE of 2489.28 and an *R*^2^ of –0.38769. TimeGPT-1 performed better with an RMSE of 1404.78 and an *R*^2^ of –0.18459.

Table 4 summarizes the performance metrics. The comparison shows that the linear regression model has the best prediction model performance with an RMSE of 1239.67, whereas the TimeGPT-1 model best fits with *R*^2^ values of –0.18459. It is important to note that this analysis uses a dataset spanning 3 years, with certain portions of the data missing. A more comprehensive X dataset and a longer time-series would likely yield improved prediction models.

**Table 4:** Performance metrics of various prediction models for time-series drug lyrics match data.

Prediction model	Training-testing ratio	Root-mean-squared error	*R* ^2^
Linear regression	2:1	1239.67	–0.91428
TimeGPT-1	2:1	1404.78	–0.18459
Facebook Prophet	2:1	2489.28	–0.38769

### A User Characteristic: The Number of Followers

We recognized that not all posts attain the same degree of exposure. The popularity of a user influences the exposure of the post. Our study includes one of the user characteristics, the number of followers of X users who posted drug lyrics. A histogram distribution method is used to extract the results. In 2015, a total of 32 users were identified, each displaying a range of followers spanning from 100,000 to 700,000.

Similarly, in 2016, a total of 14 users were found to have followers within the range of 100,000 to 1,000,000. Notably, 1 post by an influencer, with the X user ID @KameronBennett, captured attention. The post featured the line “If I Smoke this Blunt I'm Gon forget you,” extracted from the song “Love No Thotties” by Chief Keef. At the time of this post, an influencer commanded a substantial following of 1,063,400 followers. In 2017, a group of 23 users was identified, exhibiting followers ranging from 100,000 to 600,000. Another famous influencer posted, “In New York, I milly rock,” one of the most posted lines in all 3 years. The influencer had 1,093,639 followers at the time of the post. As of May 2023, he has 5.4 million followers.

## Discussion

### Principal Findings

We analyzed a dataset of publicly available posts from 2015 to 2017. The dataset comprised an extensive collection of over 1.97 billion posts, among which approximately 157 million posts exhibited a significant association with drug keywords. Within this subset, we identified 150,746 posts that specifically referenced drug lyrics. All the prediction models revealed a slight downward trend in the drug lyrics referenced on X during the time frame.

To provide further insights into the phenomenon, we examined the involvement of specific artists in generating drug lyrics on X. Our analysis contained 38 artists, where the majority of 32 (84.21%) were male, whereas the remaining 6 (15.79%) were female. The genre distribution of the quoted lyrics exhibited a marked concentration, with a significant majority (91.83%) originating from the rap, or hip**-**hop genres, (4.08%) were categorized as rhythm and blues or soul, and (4.08%) of the analyzed lyrics belonging to the pop or alternative genre.

This study analyzes drug-related lyrics in posts and identifies trends and patterns in their prevalence over time. By leveraging a dataset of over 1.9 billion posts collected between 2015 and 2017, we identified 150,746 posts containing drug lyrics. Contrary to our initial hypothesis of an increasing trend, the results indicated a slight downward trend in the frequency of drug lyrics being posted during the study period. In addition, the analysis revealed a significant association between drug lyrics and male artists, predominantly from the rap, hip-hop, and rhythm and blues genres.

The downward trend in drug lyrics posts observed in our study contradicts previous expectations and prevailing literature, which suggest an increase in drug-related content in popular media. One possible explanation could be the evolution of X’s policies or the social media landscape itself, where certain types of content may be less prevalent due to censorship or changes in user behavior. In addition, the decline may reflect a temporal shift in cultural attitudes toward drug-related content in music or perhaps even a shift toward other platforms for such expressions.

A key finding was the genre-specific nature of the drug lyrics. Rap and hip-hop artists dominated the posts containing drug-related content. This finding aligns with previous research suggesting that drug references are most prevalent in these genres, particularly in association with lifestyle themes tied to urban subcultures, personal struggles, and identity formation within these communities. The dominance of male artists also aligns with earlier studies indicating that male performers are more likely to use explicit drug references in their lyrics.

The implications of these findings are multifaceted. On the one hand, this data provides insight into the cultural and social dynamics of music consumption on social media platforms like X. It suggests that despite the pervasive influence of these lyrics, there may be a gradual decrease in their prominence, at least on X, during the time period analyzed. On the other hand, the continued prominence of drug lyrics in the rap and hip-hop genres underscores the need for ongoing dialogue about the impact of such content on young audiences and its potential contribution to normalizing drug use behaviors.

Comparing our findings with existing literature, our study supports earlier research on the association between music and drug-related behaviors but adds a new dimension by focusing on social media, specifically user-generated content on X. While much of the literature has examined the role of music in traditional media, our research shifts the focus to digital platforms, where engagement with music content is increasingly frequent.

The present study has several limitations that should be acknowledged. First, our analysis was based on a lyrics database obtained through the Genius API, which may not contain the entirety of available lyrics from other platforms, potentially limiting our findings regarding drug lyrics in popular music. In addition, we encountered unforeseen technological challenges while collecting X data streams, resulting in the absence of a portion of the data. Therefore, our conclusions depend on the available data and may not be fully generalized to the phenomenon under investigation.

One of the limitations includes artists’ use of wordplay involving drug slang, as well as other themes such as sex and violence. As previously discussed in “Popular Drug Lyrics on Twitter,” the lyrics “Double cup love, you the one I lean on” from “Best I Ever Had” by Drake is one of the examples of the artists’ wordplay. In hip-hop culture, the term “double cup” commonly denotes a styrofoam cup filled with a blend of codeine-based cough syrup and a soft drink, such as Sprite or Mountain Dew (PepsiCo). This amalgamation is closely linked to the hip-hop subculture and has been frequently cited in rap lyrics. The composition of a double cup typically includes promethazine with codeine mixed with soda and ice. The mixing process is necessitated by the potency of promethazine, which also accounts for double cup usage, as promethazine can degrade styrofoam cups if left in contact [47]. Lean, also known as Purple Drank or Sizzurp, is a concoction comprising a combination of codeine and the antihistamine promethazine, often blended with soda, cough syrup, candy, and occasionally, alcohol [48]. The evolving nature of drug slang poses one of the main challenges. Therefore, it is crucial to remain vigilant and continuously update the collection of drug keywords and slang for our post filtering purposes. Technical difficulties also posed challenges in collecting complete posts and drug lyrics from the Genius lyrics database.

Furthermore, our study focused exclusively on English-language posts, which restricts the generalizability of our findings to other languages and cultures. Identifying drug lyrics relied on traditional methods using the Smith-Waterman algorithm [40], which may introduce errors and false positives. Future research could use artificial intelligence (AI) language models, such as BERT (Bidirectional Encoder Representations from Transformers) [49] or LSTM (long short-term memory) [30], for more accurate validation and qualitative analysis to improve the identification and interpretation of drug-related content and lyrics.

Further research is needed to investigate the potential consequences of exposure to drug lyrics on X users’ attitudes, behaviors, or mental health and potential interventions or educational initiatives.

While the quantification of reposts, followers, and comments provides a metric for assessing the visibility and popularity of post content, it is essential to note that due to the real-time nature of data collection, metrics related to reposts and comments may not have reached their full maturity at the time of analysis. Therefore, our investigation primarily relies on measuring the number of followers as a proxy for evaluating the reach and potential impact of the analyzed content.

The broader implications of our study are relevant for public health and policy interventions aimed at curbing substance abuse, particularly among younger populations. By identifying specific genres and artists most associated with drug-related content, targeted educational campaigns can be developed to raise awareness about the potential influence of such content on listeners. In addition, our method of categorizing drug lyrics posts offers a novel approach for future research, providing a scalable model for analyzing social media data across various time frames and platforms. This study highlights the intersection of music, social media, and substance use and underscores the importance of continuously monitoring such trends to inform public health strategies.

Our methods are not limited to a specific time frame and can be applied to data from any period, including more recent datasets. Our approach integrates text mining, keyword filtering, and time-series forecasting models such as Facebook Prophet and TimeGPT-1, is fully adaptable to newer X data or other social media platforms. Identifying drug-related lyrics and analyzing trends is based on robust and scalable algorithms that can be updated to incorporate current data. While the present study used data from 2015 to 2017 for initial validation and analysis, our methodology remains flexible. It can seamlessly process and analyze data from more recent years, thus maintaining its relevance in ongoing research.

### Conclusions

In conclusion, our study contributes to the growing body of literature examining the interplay between popular music, social media, and drugs. It underscores the importance of understanding the cultural and societal factors influencing the dissemination and impact of drugs, ultimately aiding in the development of evidence-based strategies for prevention, intervention, and education in the context of drugs.

## Appendix

Multimedia Appendix 1 The Smith-Waterman algorithm.

Multimedia Appendix 2 The tables contain the most tweeted substance use lyrics and the associated songs for each year. The lyrics provided in the file serve as reference text, and the actual tweets may vary in spelling, word twists, and contractions or contain additional characters such as emojis.

## Abbreviations

API: application programming interface
BERT: Bidirectional Encoder Representations from Transformers
AI: artificial intelligence
LSTM: long short-term memory
RSME: root-mean-squared error

## Data Availability

The data used in this study were obtained from publicly available X feeds using the X API. The raw X data, including post IDs, timestamps, user information, and post content, were filtered based on specific criteria relevant to the study. Due to X’s data-sharing policies and privacy considerations, the raw post data cannot be shared directly. However, the processed data and the code used for data cleaning, filtering, and analysis are available upon request. Interested researchers can contact the corresponding author to obtain access to these resources. The data processing scripts, and prediction models are also available in a public repository.

## References

[1] Primack BA, Dalton MA, Carroll MV, Agarwal AA, Fine MJ (2008). Content analysis of tobacco, alcohol, and other drugs in popular music. Arch Pediatr Adolesc Med.

[2] Hall PC, West JH, Neeley S (2013). Alcohol, tobacco, and other drug references in lyrics of popular music from 1959 to 2009. Addict Res Theory.

[3] Herd D (2014). Changes in the prevalence of alcohol in rap music lyrics 1979-2009. Subst Use Misuse.

[4] Council on Communications Media (2009). From the American academy of pediatrics: policy statement--impact of music, music lyrics, and music videos on children and youth. Pediatrics.

[5] NA (2021). Every song the beatles wrote about drugs. Far Out.

[6] Strasburger VC, Hendren RL (1995). Rock music and music videos. Pediatr Ann.

[7] Hanba C, Hanba D (2018). Opioid and drug prevalence in Top 40's music: a 30 year review. J Am Board Fam Med.

[8] Jordan AE, Blackburn NA, Des Jarlais DC, Hagan H (2017). Past-year prevalence of prescription opioid misuse among those 11 to 30 years of age in the United States: a systematic review and meta-analysis. J Subst Abuse Treat.

[9] Martins SS, Sarvet A, Santaella-Tenorio J, Saha T, Grant BF, Hasin DS (2017). Changes in US lifetime heroin use and heroin use disorder: prevalence from the 2001-2002 to 2012-2013 national epidemiologic survey on alcohol and related conditions. JAMA Psychiatry.

[10] Ingraham C (2016). Heroin deaths surpass gun homicides for the first time, cdc data shows.

[11] About multiple cause of death.

[12] Hanson CL, Cannon B, Burton S, Giraud-Carrier C (2013). An exploration of social circles and prescription drug abuse through Twitter. J Med Internet Res.

[13] Morgan EM, Snelson C, Elison-Bowers P (2010). Image and video disclosure of substance use on social media websites. Comput Human Behav.

[14] Nielsen S, Barratt MJ (2009). Prescription drug misuse: is technology friend or foe?. Drug Alcohol Rev.

[15] Daniulaityte R, Carlson R, Falck R, Cameron D, Perera S, Chen L, Sheth A (2013). "I just wanted to tell you that loperamide WILL WORK": a web-based study of extra-medical use of loperamide. Drug Alcohol Depend.

[16] Daniulaityte R, Carlson R, Brigham G, Cameron D, Sheth A (2015). "Sub is a weird drug:" a web-based study of lay attitudes about use of buprenorphine to self-treat opioid withdrawal symptoms. Am J Addict.

[17] North AC, Hargreaves DJ, O'Neill SA (2000). The importance of music to adolescents. Br J Educ Psychol.

[18] Arnett J (1991). Heavy metal music and reckless behavior among adolescents. J Youth Adolesc.

[19] Stack S, Gundlach J, Reeves JL (2010). The Heavy Metal Subculture and Suicide. Suicide & Life Threat Behav.

[20] Took KJ, Weiss DS (1994). The relationship between heavy metal and rap music and adolescent turmoil: real or artifact?. Adolescence.

[21] Cranwell J, Opazo-Breton M, Britton J (2016). Adult and adolescent exposure to tobacco and alcohol content in contemporary YouTube music videos in Great Britain: a population estimate. J Epidemiol Community Health.

[22] Brown TB, Mann B, Ryder N, Subbiah M, Kaplan J, Dhariwal P, Neelakantan A, Shyam P, Sastry G, Askell A, Agarwal S, Herbert-Voss A, Krueger G, Henighan T, Child R, Ramesh A, Ziegler DM, Wu J, Winter C, Hesse C (2020). Language models are few-shot learners. ArXiv. Preprint posted online on May 28, 2020.

[23] Hong X, Sheridan S, Li D (2022). Mapping built environments from UAV imagery: a tutorial on mixed methods of deep learning and GIS. Comput Urban Sci.

[24] Li D, Jin R, Liu Z, Ren B, Gao J, Liu Z (2024). On item-sampling evaluation for recommender system. ACM Trans. Recomm. Syst.

[25] Ayranci P, Bandera C, Phan N, Jin R, Li D, Kenne D (2022). Distinguishing the effect of time spent at home during COVID-19 pandemic on the mental health of urban and suburban college students using cell phone geolocation. Int J Environ Res Public Health.

[26] Li X, Li D, Jin R, Ramnath R, Agrawal G (2022). Deep graph clustering with random-walk based scalable learning.

[27] Li D, Jin R, Liu Z, Ren B, Gao J, Liu Z (2023). Towards reliable item sampling for recommendation evaluation.

[28] Li D, Jin R, Gao J, Liu Z (2020). On sampling top-k recommendation evaluation.

[29] Press WH, Teukolsky SA, Vetterling WT, Flannery BP (2007). Numerical Recipes: The Art of Scientific Computing.

[30] Hochreiter S, Schmidhuber J (1997). Long short-term memory. Neural Comput.

[31] Lai P, NhatHai P, Han H, Anuja B, David N, Dejing D (2020). Ontology-based interpretable machine learning for textual data.

[32] Nhathai P, Manasi B, Soon AC, James G (2017). Enabling real-time drug abuse detection in tweets.

[33] Hu H, Phan N, Chun SA, Geller J, Vo H, Ye X, Jin R, Ding K, Kenne D, Dou D (2019). An insight analysis and detection of drug abuse risk behavior on Twitter with self-taught deep learning. Comput Soc Netw.

[34] Hswen Y, Zhang A, Brownstein JS (2021). Estimating the incidence of cocaine use and mortality with music lyrics about cocaine. NPJ Digit Med.

[35] Helsel P, Blankstein A (2022). Dealer sentenced to almost 11 years in rapper Mac Miller's overdose death.

[36] Romo V (2020). Juice Wrld cause of death was accidental overdose.

[37] Inkster B, Sule A (2015). Drug term trends in American hip-hop lyrics. JPMH.

[38] Miller JW Lyricsgenius: a python client for the Genius.com API.

[39] Stemming and lemmatization.

[40] Smith TF, Waterman MS (1981). Identification of common molecular subsequences. J Mol Biol.

[41] (2022). Lyricsminer source code.

[42] Forecasting at Scale.

[43] Taylor SJ, Letham B (2018). Forecasting at scale. The American Statistician.

[44] Taylor SJ, Letham B (2017). Generalized additive models for forecasting and interpreting time series data at scale.

[45] Garza A, Challu C, Mergenthaler-Canseco M (2023). TimeGPT-1. ArXiv. Preprint posted online on October 05, 2023.

[46] TimeGPT-1.

[47] What is a "double cup" in hip-hop?.

[48] Quinn D Lean Drug.

[49] Devlin J, Chang MW, Lee K, Toutanova K (2019). Bert: pre-training of deep bidirectional transformers for language understanding.

