# Supplementary material for: An Analysis of the Prevalence and Trends in Drug-Related Lyrics on Twitter (X): Quantitative Approach
Source: JMIR Form Res. 2024 Dec 30;8:e49567. doi: 10.2196/49567 (PMC11729777; doi:10.2196/49567)
Supplement: Multimedia Appendix 1 [file formative_v8i1e49567_app1.docx]

## Local Sequence Alignment (the Smith-Waterman algorithm)

## Matching a tweet to a line of lyrics is not trivial, as tweets may contain misspellings, word contractions, and twists, requiring partial or approximate matches. The approximate text-matching algorithm implemented in the LyricsMiner API is based on the Smith-Waterman algorithm, commonly employed for approximate text matching, database searches, and DNA matching. We utilized the Python implementation of fast BLAST [39] and made necessary modifications to tailor it to our requirements. Our algorithm incorporates a two-tier approach to matching sequences. Rather than comparing every character between two sets of strings, the strings are first segmented into tokens in the first tier (Algorithm 1). Then the characters within the tokens are compared in the second tier (Algorithm 2).

## The matching score is computed for each set of token alignments and propagated back to the first tier. The token match score is determined based on the alignment scores of characters in the second tier. The algorithm incorporates a gap penalty of *g = −3* for unmatched sequences. In the matrix *M*, the score for a matching cell at position *i, j* is represented as *s(M_i,j_) = 2*. The final score of the matrix *M* at position *i, j* is determined by the gap penalty *g*, the score *s(M_i,j_)*, and the scores of its three neighboring cells, as shown in Equation 1.

$M_{i, j}=\max\left\{ \begin{aligned} M_{i-1, j-1}+s\left( M_{i}, j \right), \\ M_{i-1, j}+g, \\ M_{i, j-1}+g \end{aligned} \right.$ (1)

## The alignment score decreases as the size of the gap increases. Along with the penalty imposed on unmatched sequences, the final matching score is computed as the percentage of matched sequences. The minimum achievable score is 0, while the maximum attainable score is 1.


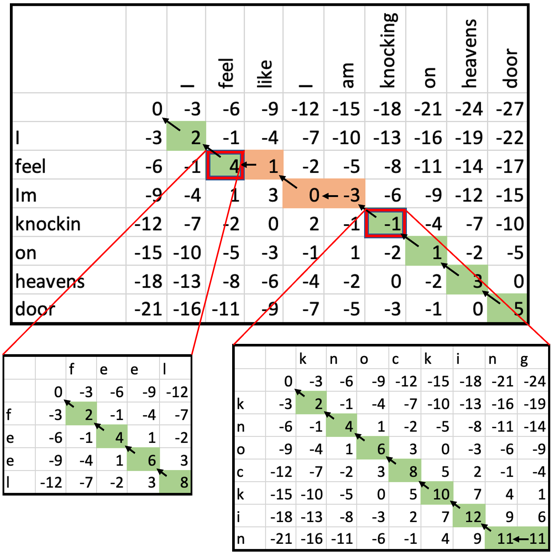


Figure 1: Two tiers text alignment; the first tier is token sequence alignment, and the second tier is character sequence alignment.


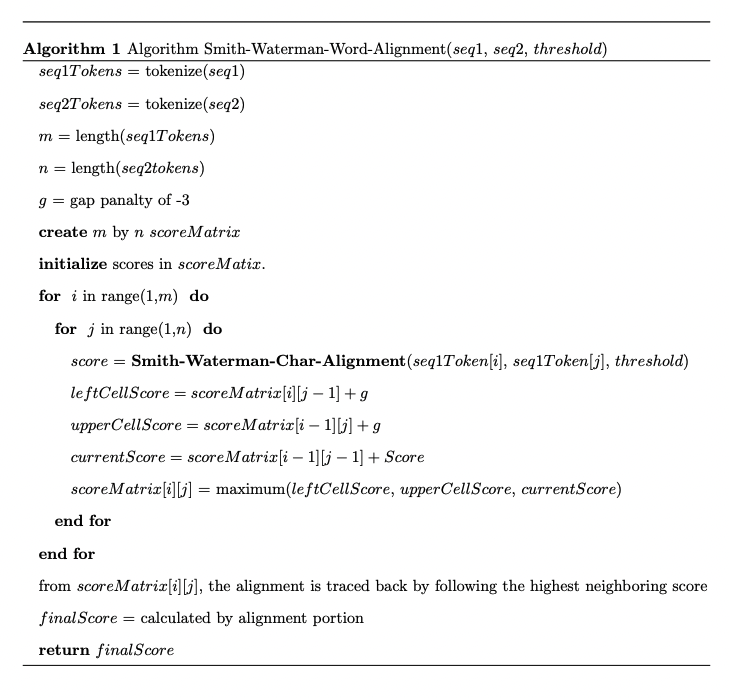


Figure 2: Algorithm 1, the first tier of text alignment.


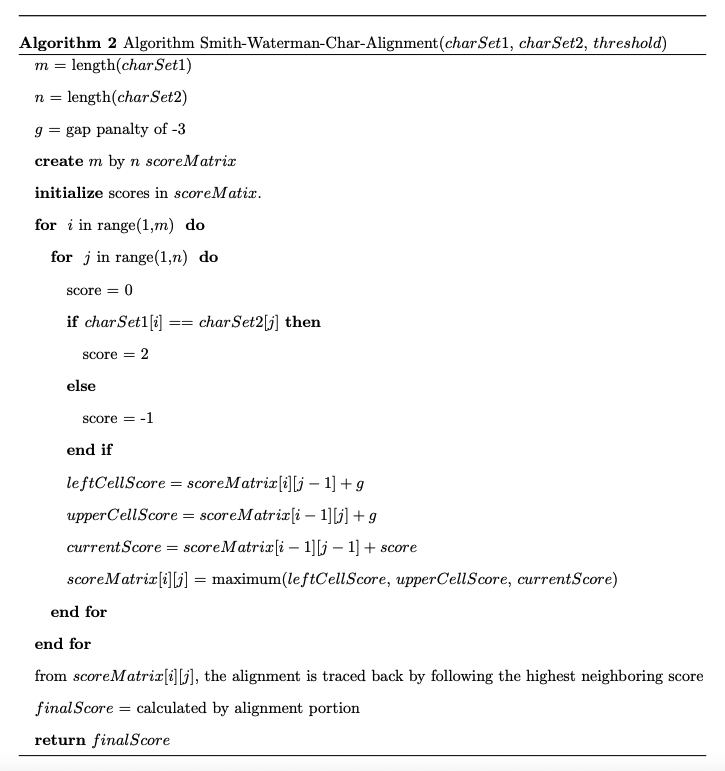


Figure 3: Algorithm 2, the second tier of text alignment.
